# Urinary neutrophil gelatinase-associated lipocalin (NGAL) might be an independent marker for anticipating scar formation in children with acute pyelonephritis

**DOI:** 10.12861/jrip.2015.09

**Published:** 2015-06-01

**Authors:** Alireza Rafiei, Hamid Mohammadjafari, Sara Bazi, Araz Mohammad Mirabi

**Affiliations:** ^1^Molecular and Cell Biology Research Center, Department of Immunology, Faculty of Medicine, Mazandaran University of Medical Sciences, Sari, Iran; ^2^Antimicrobial Resistant Nosocomial Infection Research Center, Mazandaran University of Medical Sciences, Sari, Iran; ^3^Department of Pediatrics, Faculty of Medicine, Mazandaran University of Medical Sciences, Sari, Iran; ^4^Department of Immunology, Faculty of Medicine, Mazandaran University of Medical Sciences, Sari, Iran

**Keywords:** Urinary tract infection, Scar, NGAL, Acute pyelonephritis

## Abstract

**Introduction:** Urinary tract infections (UTIs) are the most serious common bacterial infections among young children. It may affect kidneys that classified as acute pyelonephritis (APN) and may lead to renal parenchymal involvement and scarring with high prevalence rate (15%-60%) among children. This study aimed to assess the urinary concentration of neutrophil gelatinase-associated lipocalin (NGAL) in patients with APN to diagnose those with potency to scar formation.

**Patients and Methods:** Children who were admitted with a diagnosis of APN were enrolled and divided into two groups; APN with scar and APN without scar. Urinary levels of NGAL and its ratio to creatinine (Cr) levels were measured in the acute phase of infection. A receiver operating characteristic (ROC) curve was generated to allow calculation of cut-off values.

**Results:** Fifty-four children were enrolled across the 2 groups: group 1 consisted of 16 patients (all female); group 2 consisted of 38 children (36 female and 2 male). Urinary levels of NGAL were significantly higher in APN with scar than in APN without scar (*P* = 0.037). For comparison of groups 1 and 2, the cut-off values were measured as 7.32 ng/ml, sensitivity; 81.3% and specificity; 66%.

**Conclusion:** Evaluation of urinary NGAL levels may help us to identify children with APN who are at risk of developing renal scarring.

**Implication for health policy/practice/research/medical education:**

Scar formation is the most important warning in management of children with febrile urinary tract infections. Anticipating children potentially prone to scar formation in acute febrile phase, help us to better management of them. We showed here urinary levels of gelatinase-associated lipocalin (NGAL) in first days of urinary tract infection is a reliable marker to detect children prone to renal scar formation in the next future. This can be a good replacement method for Tc-99m dimercaptosuccinic acid (DMSA) if confirms by large study population.

## Introduction

Urinary tract infections (UTIs) are one of the most serious common bacterial infections among young children (1). The bacterial infections may only occur in bladder which causes lower UTI with local voiding symptoms; nonetheless, it may affect kidneys that classified as acute pyelonephritis (APN) and present with systemic and febrile symptoms. APN especially in recurrent episodes may lead to renal parenchymal involvement and scarring with high prevalence rate (15%-60%) among children (1,2). Since hypertension and end-stage renal failure might be serious consequently of renal scarring, aggressive methods used to diagnosing UTIs in the first occurrence that lead to persistent renal damage (3,4). Tc-99m dimercaptosuccinic acid (DMSA) renal scintigraphy is considered the current gold standard in imaging renal parenchymal involvement during APN and to detect late renal damage consequent to UTIs (5,6). Although DMSA scan is high sensitive method in detecting renal inflammation and scaring but it is an invasive way need to expose young children to radiation. To confirm the diagnosis of renal scar, it requires repeating DMSA 4 to 6 months later to APN (6). Moreover, DMSA could not determine children who have potential to develop persistence renal damage. Therefore introducing reliable, prompt, and definite diagnostic method to APN as early as possible is a key importance. In addition, limitation in availability of nuclear medicine departments compared to more frequently UTIs infected children, prompt scientists to search an accurate, rapid, and more practical and accessible tool that could assist pediatric clinicians in determining and managing renal parenchymal damages (7,8). The urinary biomarker levels are relatively new tools for assessing functional obstructive and other features of kidney physiological states (9,10).

Neutrophil gelatinase-associated lipocalin (NGAL), a 25 kDa protein belongs to lipocalin family, has antibacterial properties by covalently bound to matrix metalloproteinase 9 expressed by neutrophils (8). In addition to neutrophils, other cells such as epithelial and tubular cells also expressed NGAL (11). NGAL is almost low detectable under physiological conditions but rises in response to renal epithelial cells damages, the extent of this increase seems to be proportional to the severity of the endothelial damage (12). Increasing in NGAL values in the early stage of UTIs in children and renal cortical defects (13) suggesting that NGAL levels provide valuable information concerning the discrimination between the various forms of APN (14,15). These findings provide NGAL as a promising biomarker in the detection of kidney injury.

## Objectives

To the best of our knowledge to date, nothing is known about the value of urinary NGAL (uNGAL) to determining APN severe consequences, renal scaring, in young children. Therefore, we investigated urinary levels of NGAL in a collective of young children with diagnosed of UTIs and/or scaring.

## Patients and Methods

### Study population

The prospective study performed in children diagnosed with APN at Bu-Ali Sina teaching hospital, Sari, north of Iran. Before enrollment, written informed consent was given from each parents. The study population included 61 children with APN who clinically diagnosed if they had fever (core temperature ≥ 38.2°C) with or without urinary symptoms, pyuria (≥ 5 white blood cells/high power field) and positive bacterial culture. The positive urine culture was based on pure growth more than 100 000 colony forming unit of pathogen per milliliter on a urine bag or suprapubic urine specimen, more than 1000 on catheter and any colony on suprapubic sample. The urine collected by catheter or suprapubic aspiration for less than two years old patients and all the others who were unable to cooperate for urine excretion.

All patients with clinical diagnosis of APN were admitted and appropriate antibiotic was started intravenously. Third generation of cephalosporin or aminoglycoside was used for empirical therapy, upon bacterial positive culture reports, the drugs were changed according to antimicrobial sensitivity results. DMSA scintigraphic imaging was done by a (Siemens DH E-CAM) apparatus to appear renal parenchymal involvement. Inflammation was defined as attenuating in uptake in some or all portions of kidney with intact layout contour. Scar was defined as any break in kidney contour or any volume loss. Patients with any evidence of old scar in acute phase DMSA scan were excluded from study. DMSA scan was repeated 4 to 6 months later for those children with inflammatory changes. Severity of scar severity was classified as grade 1 to 4 according to international classification scaling as follows: normal=0; focal scarring in one region=1; scarring involving two regions = 2; scarring involving all three regions = 3; and generalized reduction in cortical mass = 4.

Ultrasonographic study with Siemens G-50 scanner and 2–5 MHz curved-array transducer apparatus was done for all patients. The assessment for vesicoureteral reflux (VUR) was performed for children with any abnormal findings on DMSA or US and for those with repeated APN. For diagnosing VUR, conventional voiding cystourethrography (VCUG) was performed for males and radionucleocystography (RNC) for female patients. The severity of VUR was classified on RNC as mild (equal to grade 1 and 2 on VCUG), moderate (in accordance to grade 3 on VCUG), and severe (equal to grade 4 and 5 on VCUG). For those patients who showed abnormal inflammatory changes in the first scanning, DMSA imaging was repeated 6 months after APN episode.

According to the imaging results, the patients classified into two groups; patients that whose parenchymal involvement confirmed by the second DMSA scanning considered as “APN with scar” and those with normal findings on second DMSA scan were considered as “APN without scar”. Those with previous history of APN, scar on first DMSA, or renal function impairment were excluded from the study.

### Measurement of biomarkers

All neonates were assessed for complete blood count, blood urea nitrogen, plasma and urine creatinine (Cr). Fresh voided urine samples were obtained 72 hours after starting antibiotic therapy when urine culture became negative. The samples were collected in sterile polypropylene containers. One ml aliquots were centrifuged at 4000 g for 10 minutes and the supernatant fraction stored at –80°C until analysis. Urine was tested for presence of blood or leucocytes using urine analysis strips. NGAL was determined by enzyme-linked immunosorbent assay (ELISA) kits (GenWay, San Diego, CA). Sample preparation and assay procedure were followed according to the manufacturer’s recommendation. In briefly, urine samples were placed into wells coated with affinity purified anti-lipocalin-2/NGAL antibodies as capture. After washing, anti-lipocalin-2/NGAL antibodies conjugated with horseradish peroxidase (HRP) were included. Then the enzyme bound to the immunosorbent is assayed by the addition of a chromogenic substrate, 3,3’,5,5’-tetramethylbenzidine (TMB). Reference concentrations of NGAL were used to prepare assay calibration. The absorption was determined with an ELISA reader (Biotek ELX800, USA) at 450 nm. The concentrations were interpolated from standard curves expressed in ng/ml. Inter- and intra-assay coefficients of variation were below 10%. To avoid any bias, all samples were blindly analyzed to clinical status. All samples were run in duplicate with the appropriate standards on 96-well micro plates. The results were normalized to urinary Cr values and expressed in ng/mg Cr.

### Ethical issues

The research followed the tenets of the Declaration of Helsinki; informed consent was obtained, and the research was approved by ethics committee of Mazandaran University of Medical Sciences.

### Statistical analysis

Categorical data were presented as percentages while noncategorical variables were expressed as mean ± standard deviation (SD). The normality of distribution was assessed by Kolmogorov-Smirnov test. Statistical analysis of difference between groups with normal distribution was determined by *t* test, Fisher exact test for two groups’ comparison, and chi-square for comparison of qualitative data. Nonparametric tests; Mann-Whitney U and Kruskal-Wallis tests, were used for the variables that were not distributed normally. A receiver operating characteristics (ROC) analysis was employed to calculate the area under the curve (AUC) to find the best cutoff values providing the highest diagnostic specificity followed by the best sensitivity. The results were evaluated within a 95% CI. Value of less than 0.05 considered as statistically significant. All statistical analysis was performed by using version 15 SPSS software (SPSS Institute, Chicago, USA).

## Results

### Basic characteristics

Sixty-one children were enrolled in this prospective cohort study with diagnosis of APN. Seven patients did not complete their follow up and excluded from study. The mean age of patients was 37.6±34.0 months and all but two were females. Based on the second DMSA results in 6 months follow up, the patients were categorized into two groups; APN with scar and APN without scar. As the Table 1  was shown, all APN with scar patients were females with mean age 44.1 ± 25.06 months meanwhile, 36 (94.7%) out of 38 APN without scar patients were females with mean age 34.8 ±37.2 months. Although the children in group APN without scar seems to be younger than the children with APN with scar but this difference was not met significantly (*P* = 0.367). There was no significant difference in sex, gestational maturity, or type of delivery between two groups (*P* = 0.49, *P* = 0.79, and *P* = 0.14 respectively). In addition, hemoglobin and blood urine nitrogen (BUN) concentrations were marginally increased in APN with scar group than that APN without scar group (*P* = 0.07 and 0.061, respectively). The most common cause of UTI in two groups was *E. coli*. VCUG was performed for 41 children and lead to diagnose VUR in 31 patients. The majority of APN with scar group had confirmed VUR (12 out of 15). On the other hand, 73% of APN without scar patients had VUR. There was no difference in biomarker level between patients with and without reflux (*P* = 0.46). The severity of reflux had no significant influence on urinary NGAL level (*P* = 0.31).

**Table 1:** Demographic and clinical findings of patients with APN with or without scar formation on late DMSA scan

**Parameter**	**APN with scar (n=16)**	**APN without scar (n=38)**	*** P ***
Sex			
Female	16	36	0.491
Male	0	2	
Age (months)	44.1 ± 25.06	34.8 ±37.2	0.367
Serum creatinine (mg/dl)	0.55 ± 0.16	0.6 ± 0.11	0.44
BUN (mg/dl)	24.2 ± 10.4	17 ± 5.3	0.061
Hemoglobin (g/dl)	11.2 ± 0.57	10.38 ± 1.12	0.07
VUR, n (%)			
Grade 0 (NL)	3 (20)	7 (26.9)	
Grade 1	0 (0)	4 (15.4)	
Grade 2	4 (26.7)	9 (34.6)	0.29
Grade 3	5 (33.3)	4 (15.4)	
Grade 4	1 (6.7)	0 (0)	
Grade 5	2 (13.3)	2 (7.7)	
Germ of UTI			
*E. coli*	12	30	
*Klebsiela pneumonia*	1	0	0.24
*Psudomonaaeroginosa*	0	1	

Abbreviations: DMSA, dimercaptosuccinic acid; APN, acute pyelonephritis; BUN, blood urine nitrogen; VUR, vesicoureteral reflux; NL, normal limit UTI, urinary tract infection.

### Biomarker measurements

Table 2  shows median of urinary levels of NGAL (uNGAL) and also the levels of normalized uNGAL based on Cr (uNGAL/Cr). As the urine levels of NGAL and NGAL/Cr were distributed normally, so parametric tests were performed to compare the levels of this biomarker in two study groups. The concentration of uNGAL was significantly higher in APN with scar than that APN without scar (*P* = 0.037). Meanwhile, the ratio of uNGAL/Cr was not shown significant difference in two patients groups (*P* = 0.71).

**Table 2:** Urine NGAL, creatinine and NGAL/Cr ratio in APN patients with or without scar formation

**Variable **	**APN with scar**	**APN without scar**	*** P ***
uNGAL (ng/ml)	9.8 ± 4.5	7.2 ± 3.8	0.037
Urine creatinine (mg/dl)	87.02±75.23	62.14± 63.52	0.225
uNGAL/Cr (ng/mg)	0. 22 ± 0.21	0. 24 ± 0.23	0.710

Abbreviations:‏ NGAL, neutrophil gelatinase-associated lipocalin; APN, acute pyelonephritic.

**Figure 1:**
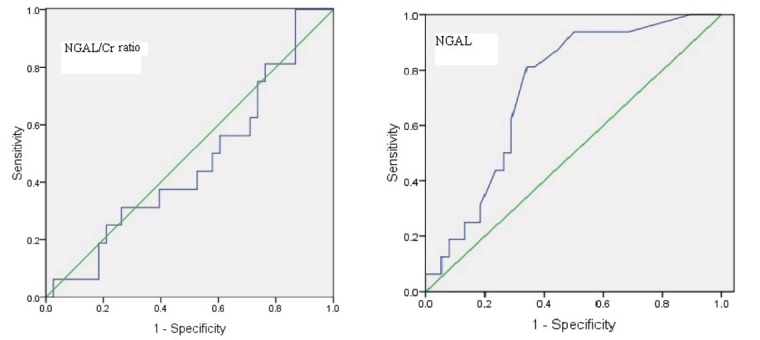
Receiver operating curves (ROC) urinary NGAL to detect scar in APN patients with and without scar. Upper curve shows urinary NGAL (area under curve [AUC] = 0.73 [SE: 0.071, 95% CI: 0.59 to 0.87]) with sensitivity 81.3% and sensitivity 0.66%. Lower curve shows ROC curve analysis for NGAL/Cr ratio; AUC = 0.47, SE: 0.09, 95% CI: 0.3 to 0.63, sensitivity 56% and specificity 0.4 ng/ml.

In order to anticipate the prognostic value of NGAL for diagnosing scar formation in APN patients, ROC analyzing was down. As Figure 1  shows the area under the curve (AUC) was 0.73 with an optimal cutoff value of 7.32 ng/ml, sensitivity = 81.3% and specificity = 66%. However, normalization of uNGAL upon Cr (NGAL/Cr) did not improve prognostic value of this biomarker in anticipating scar formation. Accordingly, the sensitivity, specificity, and AUC for uNGAL/Cr were 56%, 40%, and 0.47%, respectively.

## Discussion

Upper urinary tract infection is an important clinical challenge in pediatric field of medicine both for short term and long term morbidity. The later named renal scar should influence on children life with increasing potency to chronic renal failure, hypertension and pregnancy complications. The diagnosis of scar should be performed with a radiating study (DMSA) with at least 4 months delay (6). We assessed the urinary levels of NGAL in acute phase of APN and its relation to scar formation. Our study showed that urinary levels of NGAL are significantly higher in children with APN who developed scar on late DMSA scan.

Theoretically it is anticipated that the biomarker levels should be increased in patients with scar formation and any other kidney molecule injury. NGAL is a 25 kDa protein of the lipocalin family that was originally identified in neutrophils, but it is also expressed in kidney, liver and epithelial cells in response to various pathologic states, such as inflammation, infection, intoxication, ischemia, acute kidney injury and neoplastic transformation. At baseline only low levels of NGAL are detectable in urine. Immediately following acute kidney injury, NGAL is massively up regulated in the distal part of the nephron, i.e. thick ascending limb of Henle’s loop, distal tubule, and collecting duct (15,16). The role of NGAL in diagnosis of acute kidney injury (AKI) was widely studied in children and neonates. It was suggested that the urinary level of NGAL is significantly higher in children with tubular and intrinsic renal damage (11,17-19).

The urinary NGAL level in urinary tract infection process was assessed in recent studies (20). Yilmaz et al (21) studied sixty patients with symptomatic UTI and 29 healthy controls and measured urine NGAL by enzyme-linked immunosorbent assay. He concluded that both uNGAL and uNGAL/Cr can be used as a novel, sensitive marker for early prediction of UTI in the absence of acute kidney injury and chronic kidney disease, and the optimal cutoff value for prediction of UTI (20 ng/ml) is lower than the values determined for acute kidney injury.

Ichino et al (13), in an animal study examined renal NGAL mRNA and protein levels by real-time polymerase chain reaction. They reported that urinary NGAL levels were up-regulated in an experimental rat UTI model and they persisted at high levels even after the infection had run its course and extensive fibrosis developed. These findings suggest the potential usefulness of NGAL as a marker of renal scarring in patients with VUR in the absence of an active UTI. Yim et al (22), in a clinical study assessed 73 patients with UTI and 56 normal children and reported significantly higher levels of uNGAL (152.5 ng/ml vs. 7.5 ng/ml).

It was not aim of our study to assess the urinary level of the biomarker in children with APN. There are no published data for normal urinary biomarker levels in our region to comparing our data with normal population. The only available data belongs to study of McWilliams et al (23) in England. There are two unique studies that studied the item of scar formation. Petrovic et al (24) assessed urine and serum levels of NGAL and urinary levels of KIM-1 in 50 children with UTI and studied relation of the levels to duration of inflammation. Urinary NGAL levels in subjects with longer duration of inflammation were higher (115.37 ng/ml) than uNGAL levels in subjects with shorter duration of inflammation (67.87 ng/ml). The study focused on acute inflammatory tests for assessing the duration of infection. In our study the uNGAL levels were significantly higher in children with persistent cortical changes on DMSA scan. Therefore the criteria of persistent inflammation and fibrosis were different.

Ichino et al (25) enrolled a total of 34 patients diagnosed with vesicoureteral reflux without evidence of current urinary tract infection and 28 normal healthy children in a prospective study and reported that Urinary NGAL levels were significantly high in the VUR group but, urinary NGAL levels do not appear to reflect the severity of VUR. On the other hand the presence of renal scarring was significantly associated with higher NGAL values. They assumed the cutoff value of 1 which allowed for 89.5% sensitivity and 100% specificity for diagnosis of renal scarring. We in a similar study reached to a cutoff value of 7.315 ng/ml with a sensitivity and specificity of 81.3% and 66%, respectively.

## Conclusion

The urinary level of NGAL is a relatively suitable independent marker to predict children with APN who are prone to perform renal cortical scarring. The ratio of urinary NGAL to Cr does not predict such potency.

## Limitations of the study

The most serious limitation of our study was the relatively small sample size. This was due to both for low incidence of scar in modern medical care of pyelonephritic child and for economic limitations. We suppose to perform larger multicenter studies.

## Authors’ contribution

All authors contributed to the study. AR and HM conducted the research. AMM and SB prepared the data and prepared the primary draft. All authors read and approved the final manuscript.

## Conflicts of interest

The authors declared no competing interests.

## Ethical considerations

Ethical issues (including plagiarism, data fabrication, double publication) have been completely observed by the author.

## Funding/Support

This study was supported by a grant (#92-24) of Mazandaran University of Medical Sciences.

## References

[1] Saadeh SA, Mattoo TK (2011). Managing urinary tract infections. Pediatric Nephrology.

[2] Mohammadjafarimid  H, Rafiei  A, Abedi  M, Aalaee  A, Mirabi  Am, Abedi  E (2013). Urinary neutrophil-gelatinase associated lipocalin is a more prognosticbiomarker to distinguish antenatal hydronephrosis in neonates. Research in Molecular Medicine.

[3] Peters CA, Skoog SJ, Arant Jr BS, Copp HL, Elder JS, Hudson RG (2010). Summary of the AUA guideline on management of primary vesicoureteral reflux in children. J Urol.

[4] Jahnukainen T, Chen M, Celsi G (2005). Mechanisms of renal damage owing to infection. Pediatr Nephrol.

[5] Hoberman A, Charron M, Hickey RW, Baskin M, Kearney DH, Wald ER (2003). Imaging studies after a first febrile urinary tract infection in young children. N Engl J Med.

[6] Abedi SM, Mohammadjafari H, Hosseinimehr SJ, Mardanshahi A, Shahhosseini R (2014). Imaging of renal cortex in nuclear medicine. Journal of Clinical Excellence.

[7] Shaikh N, Ewing AL, Bhatnagar S, Hoberman A (2010). Risk of renal scarring in children with a first urinary tract infection: a systematic review. Pediatrics.

[8] Wasilewska A, Taranta-Janusz K, Dębek W, Zoch-Zwierz W, Kuroczycka-Saniutycz E (2011). KIM-1 and NGAL: new markers of obstructive nephropathy. Pediatric Nephrology.

[9] Mohammadjafari H, Rafiei A, Mousavi SA, Alaee A, Yeganeh Y (2014). Role of urinary levels of endothelin-1, monocyte chemotactic peptide-1, and N-acetyl glucosaminidase in predicting the severity of obstruction in hydronephrotic neonates. Korean J Urol.

[10] Mohammadjafari H, Rafiei A, Abedi M, Aalaee A, Abedi E (2014). The role of urinary TIMP1 and MMP9 levels in predicting vesicoureteral reflux in neonates with antenatal hydronephrosis. Pediatr Nephrol.

[11] Kuwabara T, Mori K, Mukoyama M, Kasahara M, Yokoi H, Saito Y (2009). Urinary neutrophil gelatinase-associated lipocalin levels reflect damage to glomeruli, proximal tubules, and distal nephrons. Kidney Int.

[12] Sise ME, Forster C, Singer E, Sola-Del Valle D, Hahn B, Schmidt-Ott KM (2011). Urine neutrophil gelatinase-associated lipocalin identifies unilateral and bilateral urinary tract obstruction. Nephrol Dial Transplant.

[13] Ichino M, Kuroyanagi Y, Kusaka M, Mori T, Ishikawa K, Shiroki R (2009). Increased urinary neutrophil gelatinase associated lipocalin levels in a rat model of upper urinary tract infection. J Urol.

[14] Seo WH, Nam SW, Lee EH, Je BK, Yim HE, Choi BM (2014). A rapid plasma neutrophil gelatinase-associated lipocalin assay for diagnosis of acute pyelonephritis in infants with acute febrile urinary tract infections: a preliminary study. Eur J Pediatr.

[15] Mussap M, Cibecchini F (2013). Neutrophil gelatinase associated lipocalin (NGAL): clinical significance. Ligand Assay.

[16] Singer E, Marko L, Paragas N, Barasch J, Dragun D, Müller D (2013). Neutrophil gelatinase‐associated lipocalin: pathophysiology and clinical applications. Acta Physiologica.

[17] Singer E, Elger A, Elitok S, Kettritz R, Nickolas TL, Barasch J (2011). Urinary neutrophil gelatinase-associated lipocalin distinguishes pre-renal from intrinsic renal failure and predicts outcomes. Kidney Int.

[18] Askenazi DJ, Koralkar R, Hundley HE, Montesanti A, Parwar P, Sonjara S (2012). Urine biomarkers predict acute kidney injury in newborns. J Pediatr.

[19] Askenazi DJ, Montesanti A, Hunley H, Koralkar R, Pawar P, Shuaib F (2011). Urine biomarkers predict acute kidney injury and mortality in very low birth weight infants. J Pediatr.

[20] Decavele AS, Dhondt L, De Buyzere ML, Delanghe JR (2011). Increased urinary neutrophil gelatinase associated lipocalin in urinary tract infections and leukocyturia. Clin Chem Lab Med.

[21] Yilmaz A, Sevketoglu E, Gedikbasi A, Karyagar S, Kiyak A, Mulazimoglu M (2009). Early prediction of urinary tract infection with urinary neutrophil gelatinase associated lipocalin. Pediatr Nephrol.

[22] Yim HE, Yim H, Bae ES, Woo SU, Yoo KH (2014). Predictive value of urinary and serum biomarkers in young children with febrile urinary tract infections. Pediatr Nephrol.

[23] McWilliam SJ, Antoine DJ, Sabbisetti V, Pearce RE, Jorgensen AL, Lin Y (2014). Reference intervals for urinary renal injury biomarkers KIM-1 and NGAL in healthy children. Biomark Med.

[24] Petrovic S, Bogavac-Stanojevic N, Peco-Antic A, Ivanisevic I, Kotur-Stevuljevic J, Paripovic D (2013). Clinical application neutrophil gelatinase-associated lipocalin and kidney injury molecule-1 as indicators of inflammation persistence and acute kidney injury in children with urinary tract infection. Biomed Res Int.

[25] Ichino M, Kusaka M, Kuroyanagi Y, Mori T, Morooka M, Sasaki H (2010). Urinary neutrophil-gelatinase associated lipocalin is a potential noninvasive marker for renal scarring in patients with vesicoureteral reflux. J Urol.

